# Self-reported fall and associated factors among adult people with visual impairment in Gondar, Ethiopia: a cross-sectional study

**DOI:** 10.1186/s12889-020-08628-2

**Published:** 2020-04-15

**Authors:** Moges Gashaw, Balamurugan Janakiraman, Amare Minyihun, Gashaw Jember, Kedir Sany

**Affiliations:** 1 Department of Physiotherapy, School of Medicine, College of Medicine and Health Sciences, University of Gondar comprehensive specialized hospital, University of Gondar, P.O. Box: 196, Gondar, Ethiopia; 2 Department of Health Systems and Policy, Institute of Public Health, College of Medicine and Health Sciences, University of Gondar, Gondar, Ethiopia

**Keywords:** Fall, Visual impairment, Medications, Depression, Fear of fall, Ethiopia

## Abstract

**Background:**

Fall is a major public health problem and potentially disabling issue. A vast burden of visually impaired live in low-middle income countries particularly in Sub-Saharan Africa. Limited ability to detect environmental hazards puts visually impaired at a greater risk of falls and unintentional injuries. Falls among visually impaired is associated with considerable disability, health care cost, loss of independence, and socio-economic consequences. Ethiopia lacked estimates of fall among any vulnerable population, particularly among visually impaired people. Therefore, this study aimed to estimate the prevalence of falls and factors associated among adult people with medically diagnosed visual impairment in Ethiopia.

**Methods:**

An institutional-based cross-sectional study was conducted among visually impaired adults who attended the ophthalmology clinic at the University of Gondar comprehensive specialized hospital during the study period. Data were collected by interview method using structured questionnaires, patient medical record reviews, and physical measurements. Bivariate and multivariable binary logistic regression model analysis was used to identify factors associated with falls. Adjusted odds ratio with corresponding 95% confidence intervals were computed to show the strength of association.

**Results:**

A total of 328 adults medically diagnosed with visual impairment participated in the study (97.3% response rate). The age of the participants ranged from 25 to 89 years with a mean age of (56.46 ± 14.2 years). The overall cumulative prevalence of self-reported falls among adults with visual impairment was 26.8% with 95%CI (22.7, 32.4%). The major associated factors of fall identified by multivariate analysis were; visual impairment in both eye (AOR 3.21, 95% CI 1.11, 9.29), fear of falling: some concerned: (AOR, 4.12; 95%CI, 1.44, 11.76), very concerned fear (AOR 10.03; 95% CI, 3.03, 33.21), medications: (AOR 4.63; 95% CI 2.14, 10.00) and self-reported depression: (AOR 3.46; 95% CI 1.11, 10.79).

**Conclusion:**

The result of this study indicates a moderate self-reported prevalence of fall among adult people with medically diagnosed visual impairment. Identifying sub-groups at risk of falls among visually impaired, modifiable risk factors, implementation of precaution measures to avoid fall and fall-related injuries, and most importantly measures that would reduce the fear of falls in visually impaired people deserves immediate attention.

## Background

Falls are a major public health problem and an estimated 646,000 fatal falls occur each year. It is also one of the leading causes of unintentional injury-related death and can cause substantial morbidity [1, 2]. Visual impairment is reported to be an independent risk factor for falls and fall-related death among adults aged over 50 years [3]. Fear of falling that precede or results from falls is a distinct health outcome and the process can sometimes become a vicious circle. Fear of falling is a low perceived ability to avoid falling while performing routine actives of daily living and has been associated with self-imposed restrictions in physical activity and social participation [4, 5].

Visual impairments reduce one’s ability to detect hazards in the environment and have a drastic impact on the physical, psychological, and socio-economically development of individuals and societies [6]. Visual impairment is more prevalent in low and middle-income countries (LMICs), about 80–90% of the world’s visually impaired people live in LMICs [7]. In Sub-Saharan African countries, about 70 million people had been reported to live with visual impairment and Ethiopia is believed to have one of the world’s highest rates of blindness and low vision, of which about 80% is preventable or treatable [8, 9].

Studies had reported a wide range of risk factors for fall; age, gender, severity of visual impairment, body mass index, psychosocial, physical environment, fear of fall, depression, decreased vestibular reflexes, decreased muscle strength, increasing postural sway, concurrent medical issues, poly-medications, and pre-existing medical co-morbidities [2, 10, 11]. Polypharmacy is also one of the important risk factor for falling and the association is stronger if the medications include fall risk-increasing medications [12].

In particular, fear of fall among people with eye diseases has been related to higher chances of falls. Fear of fall can lead to loss of confidence, depression, activity avoidance, reduced social interaction, and disability. Therefore, fear of falling is a key variable in order to understand factors with fall risks and for the development of prevention strategies [13–15].

Falls among visually impaired adults can occur due to multifaceted intrinsic and extrinsic factors. In LMICs people with visual impairment are confronting a different set of challenges like accessibility barriers, navigator barriers, unemployment, health care utility challenges, and societal stigma [16, 17]. Though, population-based studies had reported that old age and related-poor vision as a frequent risk factor for fall, yet the knowledge about visual-related clinical characteristics in falls among adults living in LMICs is sparse. Estimates of falls and their predictors are largely unexplored in any population in Ethiopia and more importantly in the visually impaired population who are at a greater risk of falls and related morbidity. Hence, to gain an understanding of the factors associated with this population living in LMICs like Ethiopia is crucial. This study is a preliminary attempt to determine the burden of falls, and associated factors among adult people living with visual impairment in Ethiopia. Thus this evidence will help us to establish preventive measures among this population in Ethiopia.

## Methods

### Study design, setting, and participants

An institutional-based cross-sectional study was conducted from January to June 2018 among adults with medically diagnosed visual impairment who seek care from the ophthalmology clinic at the University of Gondar comprehensive specialized hospital (UoGCSH), North West Ethiopia during the study period. This eye care center provides a comprehensive clinical and community eye health services free of cost for eight zones and serves as a major referral center for 14 million people living in and around Gondar, North West Ethiopia. It is estimated that a minimum of 4720 patients utilizes different eye care service per year and this is the only clinical catchment area for visually impairment individuals in Gondar city. Based on the 2016 population estimate of Gondar city administration bureau had a total population of 335,000. The study area is geographically a challenging mountainous terrain and unsafe sometimes even for normal-sighted people and hazardous for susceptible people like older, physically challenged and people with visual impairment. The study setting (UoGCSH), the ophthalmic outpatient clinic receives a wide range of ambulatory VI patients visiting for the consultation, laboratory testing, medications, eye-glasses, mobility aids, and surgery appointments and the study setting is the only referral hospital for the entire Gondar city. Hence, individuals with a wide spectrum of visual impairment visit this institution. As of 2017, the ophthalmic clinic cares for 40 to 60 visually impaired patients every day. So, an average of 1500 adult participants is expected to visit the clinic during the 6 weeks data collection period.

Adults with medically diagnosed visual impairment in one or both eyes, those who can walk independently with or without mobility aids, aged ≥18 years with hearing ability were included in this study.

### Sample size determination and sampling procedure

The sample size required for this study was determined using the single population proportion formula [18] and calculated using Epi Info software version7.0 (Centres for Disease Control and Prevention, USA). The samples were obtained from a relatively small population (*N* = 1500). The following assumptions were used to determine the required sample: prevalence of 50% since no past regional data exists, a confidence level of 95, 5% margin of error. The derived power calculated sample size was *n* = 306. Accounting for an estimated refusal or non-response rate of 10%, the final sample size was calculated to be *n* = 337.

The study participants were recruited by using a systematic random sampling method by arranging the patients based on their chart number from the registered appointment register selected in K^th^ interval each day during the study period. The first patient between 1 and K was selected randomly by a lottery method, and the next patient was interviewed every fourth interval. The procedure was repeated until the estimated eligible sample size was reached.

### The operational definition

Self-reported fall in the past12 months was defined as an event that results in a person coming to rest unintentionally on the ground or other lower level, not a result of acute illness, at least once in the past 12 months [19]. Fear of fall was assessed by close-ended a question phrased as: “Are you afraid or concerned about falling while performing activities of daily living (ADL)? ” [20]. Visual impairment (in any eye); significant loss of vision on which Snellen’s chart reading is less than 6/12 to no light perception, Mild visual impairment; presenting with a visual acuity of less than 6/12(20/40) greater than or equal to 6/18(20/60). Moderate visual impairment; presenting with visual acuity of less than 6/18(20/60) to greater than or equal to 6/60(20/200) and severe visual impairment is presenting with visual acuity of less than 6/60(20/200) to no light perception. Visual impairment, which could not be eliminated by refractive correction or lenses (non-correctable) [8].

### Study procedure

This cross-sectional study was approved by the Gondar University School of Medicine research and ethical review committee (SOM 2310706). After providing a verbal account and explaining about this study to the participants with visual impairment, the written informed consent statement was obtained. A structured data collection questionnaire was developed based on an extensive review of related studies [21–24]. The questionnaire was initially prepared in English and then translated to Amharic and back to English by language experts at University of Gondar and checked for the consistency of the questions and corrections were made accordingly.

The questionnaire included domains like socio-demographic characteristics: age, gender, behavioral characteristics: physical measurements, visual related characteristics, medical co-morbidity, and fall-related characteristics including fall-related injuries (Additional file 1). A review of the participant’s hospital records for laboratory investigations results, medications, and medical illnesses were done. And thereafter, the selected participant was referred to the ophthalmology clinic for visual assessment. The questionnaires were orally administered to each study participant individually during the interview by four trained ophthalmic nurses and the responses of the patient were recorded in the data collection sheet. Factors related to visual impairment, visual acuity measurement, Snellen’s E chart measurement, and medical eye screening reports evaluated by the optometrist on the day of data collection was recorded by the data collector.

Counter checking of the daily filled questionnaires and regular supervision was done by MG and BJ. The questions were read aloud and the participant’s responses were recorded. To avoid any bias that may have affected the participants’ responses the questions were read for each participant individually in a quiet room and they were told to repeat the questions again to the data collector to assure the reception of questions clearly. The data collectors made sure that the questions were simplified as much as possible, caregivers were accompanied during self-reporting, and explanations were given whenever a question aroused.

### Data processing and analysis

Data were checked for completeness and entered into Epi-Info version 7.1 and then exported to IBM SPSS version 20 statistical software for coding, recoding, storing and further analysis. Descriptive statistics like frequencies, percentages, means, and standard deviations were used for all participant characteristics and factors associated with falls. With self-reported falls in the past 12 months (categorized as none versus present) as the dependent variable, logistic regression analyses were done to determine the association with different independent variables. Independent variables included in the regression models were socio-demographic characteristics: age in years (categorized: adults 18–44, older adults 45–64, and elders > 64), gender, residence (categorized urban, rural), marital status (categorized not married, married, divorced, widowed), level of education (categorized no formal school, primary, secondary, diploma and above), occupation (categorized homemaker, farmer, civil servant, merchant, retired, others), income (categorized < 1000,1000–2000, > 2000- < 3000, > 3000 in Ethiopian birr per month), physical measurements like height, weight, BMI (categorized: underweight, normal weight, overweight, and obese), behavioral factors: alcohol drinking (categorized never, past, current), smoking (categorized never, past, current), self-reported regular physical exercise (none, present). Psychosocial factors: fear of falling (categorized not at all concerned, somewhat concerned, and very concerned), social support (yes, no), depression (yes, no). Vision and health-related factors: mild vision impairment, moderate vision impairment, severe vision impairment, and eye involved (one versus both), current level of mobility, comorbid, drug in-take, place of fall, and activity during fall.

Prior to conducting the regression model the following assumptions were tested: multicollinearity (Variance inflation factor < 10) independence of residuals by scatter plots. Initially, bivariate analysis was conducted and independent variables that were found statistically significant were fitted into the multivariate model using the backward stepwise (likelihood ratio) method.

We tested for potential statistically significant interactions by adding the product of the covariates in the multivariable-adjusted logistic regression models. Crude and adjusted odds ratios and 95% confidence intervals were calculated from univariate and multivariate logistic models for associations between the independent variables and the dependent variable. Variables were entered into the model using forced entry and categories were used as covariates for detailed analyses. Results were considered statistically significant when 95% confidence intervals not containing unity (*p*-value < 0.05) for both main effects and interaction terms. Interaction terms were used to examine the potential association between fear of fall and fall differed by hypothesized variables, including age category, the severity of visual impairment, depression, eye involved, and medication intake. When clear sub-groups seemed to be present in the dataset, significance testing (Pearson χ^2^) and, if appropriately sized subgroups per category remained, logistic regression analyses were conducted. Finally, the research was checked for the adherence to STROBE guidelines (Additional file 2).

## Results

### Socio-demographic and behavioral characteristics

A total of 337 participants were approached for participation. Among those 328 visually impaired adults consented to participate and completed the questionnaire with a response rate of 97.3%. This is more than 100% of the power calculated sample size (*n* = 307). The reasons for non-responses were no time, not interested, unable to find their medical chart, and refusal to take visual acuity tests. The main socio-demographic characteristics of adults living with visual impairment in Gondar are shown in Table 1. The mean age of respondents was 56.46 (SD ±14.2) years and their age ranged from 25 to 89 years. Nearly, two-thirds of them (67.7%) were in the age group 64 years and below. Among the 328 participants, the majority of them 189 (57.6%) were males. More than half (59.1%) of them were uneducated, almost four in five (79.6%) were married, about two in five (42.2%) had a monthly income of less than 1000 birr, and most of them (61.3%) reported to have a good family or social support. About 70% of the participant’s BMI category was normal weight followed by underweight (8.9%). Only a few (13.1%) reported being involved in physical exercise, a minority of them (10%) were current smokers, and most of them (60.7) never consumed alcohol.

**Table 1:** Socio-demographic characteristics of adults living with visual impairment in Gondar, University Specialized Referral Hospital, Ethiopia (*n* = 328)

Variables	Frequency	Percent (%)
Sex		
Male	189	57.6
Female	139	42.4
Age in years (mean age 56.46 ± 14.2)		
18–44	78	23.8
45–64	144	43.9
> 64	106	31.5
Residence		
Urban	135	41.2
Rural	193	58.8
Marital status		
Not married	10	3.0
Married	261	79.6
Divorced	31	9.5
Widowed	26	7.9
Religion		
Orthodox	296	90.3
Protestants	3	0.9
Muslims	29	8.8
BMI (kg/m^2^) (mean 19.94 ± 2.15)		
Underweight (< 18.5)	29	8.9
Normal (18.5–24.9)	231	70.4
Overweight (25–29.9)	13	3.9
Obese (> 29.9)	5	1.8
Occupation		
Homemaker	99	30.2
Farmer	145	44.2
Civil servant	29	8.8
Merchant	32	9.8
Retired	16	4.9
Others^a^	7	2.1
Level of education		
No formal school	194	59.1
Primary school	71	21.6
Secondary school	27	8.2
Diploma	17	5.2
Degree and above	19	5.8
Income (ETB/month)		
< 1000	140	42.7
1000–2000	104	31.7
2001–3000	46	14.0
> 3000	38	11.6
Family/social support		
Yes	201	61.3
No	127	38.7
Smoking habit		
Never	313	95.4
Past smoker	5	1.5
Current smoker	10	3
Drinking alcohol habit		
Never	199	60.7
Past alcoholic	39	11.9
Current alcoholic	90	27.4
Physical exercise		
Yes	43	13.1
No	285	86.9

^a^Daily labor workers, students, *BMI* body mass Index, *ETB* Ethiopian birr

### Vision specific characteristics of the participants

Among the total study subjects, more than one third 133 (40.5%) of the patients were diagnosed with mild visually impaired based on Snell’s E-chart reading. Of all the participants, 63(19.3%) were diagnosed with severe visual impairment with presenting visual acuity (< 6/60 to no light perception). Table 2 shows the vision-specific characteristics of the participants. The most common cause of visual impairments among the study participants was glaucoma (46.6%) followed by refractive errors (19.3%).

**Table 2:** Vision specific characteristics of the participant in Gondar University Referral Hospital, Ethiopia (*n* = 328)

Variables	Frequency(n)	Percent (%)
Cause of visual impairment		
Cataract	34	10.4
Glaucoma	153	46.6
ARM	25	7.6
Diabetic retinopathy	13	4.0
URE	59	18.0
Others eye disease	44	13.4
Severity of visual impairment		
Mild VI	133	40.5
Moderate VI	132	40.2
Severe VI	63	19.3

*ARM* age-related maculopathy, *URE* uncorrected refractive error, *VI* visual impairment

### Prevalence and distribution of fall among adults with visual impairment

The prevalence and distribution of falls among adults with visual impairment are shown in Table 3. Eighty-eight (26.8, 95% CI: 22.7, 32.4%) visually impaired patients reported to have experienced at least one fall in the previous 12 months. Among those fallers, 14.8% of them reported having fallen more than once with the mean of 1.49 (±1.42) falls among 88 fallers. The maximum number of falls reported was 7 times by 3 participants. The prevalence of fall was nearly equal for male (52.3%, *n* = 46) as for female (47.7%, *n* = 42) participants. A significant difference was observed in previous 12 months self-reported fall prevalence between categories of fear of fall (not concerned 6.5%, somewhat concerned 24.3%, and very concerned 53.5%; χ^2^ (2, *n* = 328) = 72.9, *p* < 0.001), phi = 0.47). Among the fallers, 19.1% of them reported having used some assistive mobility devices at the time of fall. Majority of the study participants were diagnosed with visual impairment in both their eyes and prevalence of fall was vastly different (one eye 7.9% versus both eyes 32.5%; χ^2^ (1, n = 328) = 18.1, p < 0.001), phi = 0.23). The highest prevalence of fall was reported by adults those who are diagnosed with glaucoma 37(42.1%) followed by cataract 21(23.8%). Among the participant who reported fall, most of them 63(70.8%) had suffered one or more fall-related injuries.

**Table 3:** The self-reported prevalence of fall among adults with visual impairment in Gondar University Specialized Referral Hospital, Ethiopia

Variables	Self-reported falls	
	Yes (***n*** = 88)	No (***n*** = 240)
Sex		
Male	46 (52.3)	143 (59.6)
Female	42 (47.7)	97 (40.4)
Residence		
Urban	45 (51.1%)	90 (37.5)
Rural	43 (48.9%)	150 (63.5)
Age in years		
18–44	16 (18.2)	62 (30.2)
45–64	34 (38.6)	110 (45.8)
> 64	38 (43.2)	68 (28.3)
BMI in kg/m^2^		
Underweight (< 18.5)	27 (30.7)	52 (21.7)
Normal (18.5–24.9)	55 (62.5)	176 (73.3)
Overweight (25–29.9)	6 (6.8)	7 (2.9)
Marital status		
Single not married	4 (4.5)	8 (3.3)
Married	60 (68.2)	201 (83.7)
Divorced	8 (9.1)	23 (9.7)
Widowed	16 (18.2)	8 (3.3)
Fear of fall		
Not concerned	8 (9.1)	115 (47.9)
Somewhat concerned	26 (29.5)	81 (33.7)
Very concerned	54 (61.4)	47 (19.4)
Severity of impairment		
Mild visual impairment	18 (20.4)	115 (47.9)
Moderate visual impairment	39 (43.3)	93 (38.7)
Severe visual impairment	31 (35.3)	32 (13.3)
Eye involved		
One eye	06 (6.9)	70 (29.2)
Both eyes	82 (93.1)	170 (70.8)
Cause of Visual impairment		
Cataract	21 (23.9)	13 (6.7)
Glaucoma	37 (42.0)	116 (59.8)
ARM	8 (9.1)	17 (8.8)
Diabetic retinopathy	10 (11.4)	3 (1.5)
URE	8 (9.1)	5 (2.6)
Others eye disease	4 (4.5)	40 (20.6)
Level of mobility		
Independent without mobility aid	56 (63.6)	209 (87.1)
Independent with mobility aid	32 (36.4)	31 (12.9)

*BMI* body mass index, *ARM* age-related maculopathy, *URE* uncorrected refractive error

### Factors associated with self-reported fall

Table 4 shows the association between socio-demographic, psycho-social, visual related factors, clinical characteristics, and fall among the study participants. Fear of fall among the visually impaired participants was one of the main variables associated with fall in the regression model. Those participants who had reported that they are somewhat concerned about the fear of falling during ADL were 4.12 times more likely to fall as compared to participants not concerned fear of falling (AOR 4.12; 95% CI, 1.44–11.76), The odds increase by 10.03 (95%CI, 3.03–33.21) if the participants reported to very worried or concerned about fall during ADL compared to those participants who had no concern about the fear of falling. The adjusted odds of fall were 4.63 times higher among people who had taken one or more medications as compared to their counterparts (AOR 4.63; 95% CI 2.14–10.00). Participants with visual impairment in both their eyes were more than 3 times more likely to sustain fall than those with unilateral visual impairment (AOR 3.21, 95% CI 1.11–9.29).

**Table 4:** Factors associated with fall among adults with visual impairment in Gondar University Specialized Referral Hospital, Ethiopia (n = 328)

Variables	Self-reported fall		Univariate COR (95%CI)	Multivariate AOR (95%CI)
	Yes	No		
**Age (years)**				
18–44	16	62	1.19 (0.61–2.34)	0.39 (0.14–1.16)
45–64	34	110	2.17 (1.09–4.27) *	0.37 (0.12–1.16)
> 64	38	68	1.00	1.00
**Residence**				
Urban	45	90	1.00	1.00
Rural	43	150	0.57 (0.35–0.94) *	0.69 (0.32–1.49)
**Fear of falling**				
Not at all concerned	8	115	1.00	1.00
Somewhat concerned	26	81	4.49 (1.94–10.43) **	4.12 (1.44–11.76) **
Very concerned	54	47	16.08 (7.11–36.4) **	**10.03 (3.03–33.21**) **
**Sleep disturbance**				
No	48	203	1.00	1.00
Yes	40	37	4.57 (2.67–7.89) *	1.62 (0.73–3.58)
**Depression**				
No	76	230	1.00	1.00
Yes	12	10	3.6 (1.51–8.74) **	3.46 (1.11–10.79) **
**Severity of visual impairment**				
Mild	18	115	1.00	1.00
Moderate	39	93	0.16 (0.08–0.33) *	1.01 (0.32–3.14)
Severe	31	32	0.43 (0.23–0.79) *	1.11 (0.47–2.63)
**Visual impairment**				
One eye	06	70	1.00	1.00
Both eyes	82	170	5.62 (2.35–13.49)*	3.21 (1.11–9.29)*
**Medication intake**				
No	32	190	1.00	1.00
Yes	56	50	6.65 (3.89–11.35) **	4.63 (2.14–10.00) **
**Diabetes mellitus**				
No	74	234	1.00	1.00
Yes	14	6	7.38 (2.42–19.88) *	1.64 (0.24–10.79)
**Hypertension**				
No	72	231	1.00	1.00
Yes	16	9	5.7 (2.42–13.45) *	1.85 (0.55–6.17)

*variables significant with *p*-value ≤0.01; ** variables significant with *p*-value ≤0.05, 1 = reference category; *COR* crude odds ratio, *AOR* adjusted odds ratio, *CI* confidence interval

Visually impaired people who had depression were 3.46 times more likely to develop fall as compared to their counterparts (AOR 3.46; 95% CI 1.11–10.79). The interaction effect between age and severity of visual impairment, age, and eye involved was not significant. Moreover, the causes of visual impairment were not associated (*p* < 0.2) with the outcome variable in the univariate logistic model (Additional file 3).

## Discussion

The findings of this study showed that the overall prevalence of fall among visually impaired adult people was 26.8% with 95% CI (22.0–31.4). This implies that falls are a major public health problem, common reason for hospital trips in fall-related fractures, injuries (Table 2 in Additional file 3) and one of the main causes of disability among visually impaired people. This finding is harmonized with the studies conducted in Salisbury city in Maryland, USA, 28.9 and 27.8% respectively [25, 26], Cape Town, South Africa, 26% [27] and Ibadan, Nigeria 23% [28]. This study found that the prevalence of fall among visually impaired adults increased with an increase in age, for age groups 18–44 years, 45–64 years and above 64 years the prevalence of fall in the past 12 months was 18.2, 38.6, and 43.2% respectively. Similarly, the study conducted in Latino, USA reported the prevalence of fall to be 18% in the age group 18–44 years age, 21% in 45–64 years and 35% in older adults age greater than 64 years [2]. This could be due to physiological change related to age, when the age increases the severity of visual impairment also increases, muscle and bone become deteriorated and weak so easily vulnerable for fall and fall-related injuries [29]. Surprisingly, in this study, there is neither association between fall and the severity of visual impairment nor correlation between the number of falls and severity of visual impairment, which is contrary to the findings elsewhere [30–32]. The possible reasons might be that the study area (UOGCSH) is the only public ophthalmic clinic for the region and receives patients with a wide spectrum of eye diseases, from less severe to more severe. Besides, self-imposed restrictions and low level of mobility among participants with severe visual impairments could be other reasons for limited exposure and lesser falls. More notably, in LMICs like Ethiopia, people with visual disabilities face prejudice, stigma, limited job opportunities, inconvenient infra-structure, and lesser chance of exploring the environment.

However, the prevalence rate reported in this study is found to be lower when compared to studies conducted in USA 57.9% [33] and Sweden 38% [34]. This difference might be due to the difference in the study area, methodology, level of mobility of the participants, and the severity of visual impairments in the participant. The Sweden study was a community-based survey, the majority of their participants suffered a mild visual impairment and the mean age of their participants was much higher compared to this study probably due to higher life expectancy in the developed countries. In contrast, this study was institutional-based design, the mean age of our sample is much lesser, there is a difference in sample size, and variability in participant socio-demographic characteristics which might be the possible reasons for the difference in the estimates.

However, this finding is higher than other studies conducted elsewhere, in Malay, Singapore 14.7% [35] in blue mountain eye study, Australia 16.1% [31], and a prospective longitudinal study in Hong Kong, China 19.3% [36]. The possible reason for this discrepancy might be the difference in sample size, study design, methods, infra-structural barriers in low-income countries, geographical terrain challenges of the study area, and economical and lifestyle variation. In particular, factors like difference in the educational level of the participants, availability of mobility aids, awareness program on risk factors and preventions, adjunct care in the eye clinic, and health-related facilities might also be the reasons for lower prevalence in these countries.

Another notable finding is that the fear of falling was an independent predictor of fall with higher odds of fall. It is also known that an actual fall leads to fear of falling syndrome and vice-versa [26]. This finding was supported by Salisbury, Maryland study [25]. This also suggests that fear of falling is not just an acute outcome that results from a fall; rather, it is likely recognition of being at risk and fear of falling leads to fall and vice-versa. This could be due to the reasons that fear of falling leads to self-imposed restrictions like activity limitation, isolation, decrease the quality of life of the individual, loss of confidence, depression, result institutionalization and decreased physiological function leads to a high risk of fall and falling [14]. Moreover, fear of fall has gained acceptance as a very disabling medical issue and is present even in those who had never fallen before as a result of anticipatory anxiety. Thus, fear of falling shall be accounted for as one of the significant predictors of future falls.

Taking medications was also found to be one of the predictors of fall among visual impairment people in this study and should have more obvious implications for primary prevention strategies. This finding was supported by the study done in Salisbury, Maryland [25], and another cross-sectional study found that medication was significantly associated with fall among older adults [36]. The adverse effect of the medications, drug-drug interaction, metabolic effect of the drug on the body, and some antidepressants and antipsychotics can also cause fall secondary to orthostatic hypotension. In addition, those class of drugs that reduces the blood pressure or slows the heart can also contribute to falls [37, 38].

This study also showed that depression is associated with a two-fold higher risk of fall among visually impaired adults. Actual fall or fear of fall, visual impairment, and self-imposed mobility limitations could result in depression rather than depression being a casual or risk factor. The findings also suggest that depression prevention and treatment are a much-needed step towards fall prevention intervention [3, 39]. Moreover, there seems to be a web of causation between actual fall, fear of fall, and depression among visually impaired individuals.

Given that people with visual impairment in this study are at risk of fall and fear of fall, the findings emphasize the importance of addressing falls and fear of falling in this susceptible group. For the benefit of future researches, there are some noteworthy limitations. First, falls were self-reported with a recall period of 12 months and the potential recall bias was minimized by excluding participants with mild cognitive impairment. Second, confounders, unmeasured or unknown factors (societal or lifestyle) could have an influence on the findings. The cross-sectional nature of this study presents limitations in terms of causal association interpretations. For example, this study demonstrated the possible endogenous effect between fear of falling and falls. But the casual direction between these two variables cannot be explained. Other possible risk factors of fall like impaired muscle strength, vestibular, and postural impairments were not measured. The majority of the participants in this study could not recall the exact duration of visual impairment since diagnosis and therefore the association between adaptation to visual impairment and risk of fall is not known. Nevertheless, no prior study has directly examined the prevalence of fall in visually impaired people in Ethiopia and we strongly feel that these findings will provide more insight into the burden of fall in Ethiopia.

## Conclusions

In summary, our findings from an adequately powered sample of visually impaired adults of Ethiopia document independent relationships between fear of fall, medication, depression, and falls in the past months. Other factors and confounders that may help explain fall in visually impaired adults, and the interaction between falls, fear of falling, loss of mobility, quality of life, and loss of confidence should be explored in future studies. Because each is a risk factor for the other, a person who has one of these factors is at risk for developing the other one. Fear of falling among visually impaired or any vulnerable individuals deserves special attention. In the meantime, health advocates and health policy providers are recommended to develop fall risk assessment, environment inspection, and modification, identify individuals at risk to educate them and provide fall-prevention treatments to avoid future fall and fall-related injuries.

## Supplementary information

**Additional file 1.** English version questionnaire.

**Additional file 2.** STROBE statement checklist.

**Additional file 3.** Additional table for univariate analysis of visual characteristics and fall, frequency distribution of fall-related injuries.

## Data Availability

Data will be available upon formal request from the corresponding author.

## Abbreviations

ADL: Activities of daily living
AOR: Adjusted odds ratio
BMI: Body mass index
CI: Confidence interval
COR: Crude odds ratio
CSA: Central Statistical Agency
CVD: Cardio-vascular disease
DM: Diabetes’ Mellitus
FMOH: Federal Ministry of Health
HTN: Hypertension
LMIC’s: Lower and Middle-Income Countries
OR: Odds ratio
TBI: Traumatic brain injuries
UOGH: University of Gondar Hospital
USA: United State of America
VI: Visual Impairment
WHO: World Health Organization
