# Supplementary material for: Self-reported fall and associated factors among adult people with visual impairment in Gondar, Ethiopia: a cross-sectional study
Source: BMC Public Health. 2020 Apr 15;20:498. doi: 10.1186/s12889-020-08628-2 (PMC7161228; doi:10.1186/s12889-020-08628-2)
Supplement: Supplementary file 1 — Additional file 1. English version questionnaire. [file 12889_2020_8628_MOESM1_ESM.docx]

# Appendix -I

## Annex I. Consent form and information sheet

Dears, Data collectors, please read the following statement to the respondents before you administer the interview/questionnaire

Dear Participants:

Good morning/afternoon? I am ……………………….. And I am one of the data collectors on behalf of the investigator

The University of Gondar and Principal Investigator are jointly conducting this study in UOG. This study aimed at better understanding of the “the prevalence of fall and associated risk factors among visual impairment adult people attending in university of Gondar specialized hospital.Gondar,North West Ethiopia.

You have been chosen to participate in this study. The purpose of this questionnaire and assessment is to find out the information about falls among visually impaired adult people, in addition to the questionnaire; we will measure your height and weight to know your BMI.

**Procedure:** In order to collect our data, we invite you to take part in our project. The permission will be possessed on university of Gondar. If you are willing, you need to understand and sign the consent form.

**Risk and /or discomfort:** The participant will not face any risk or discomfort by being participating in this study except that he/she delicate his /her time to respond to the questions. Any participant’s response will not be copied or transferred to another body. Every piece of information will be kept confidentially.

**Benefits:** If you are participating in this research project, the output of the study will have indirect benefit to you, as well as the community at large in the future.

**Incentives/payments for participating:** There will not be any incentive or payment for the participants by being participating in the study.

**Confidentiality:** The information collected from this research project will be kept confidential and information about you that will be collected by this study will be stored in a file, without your name, The information collected from this research project will be kept confidential and the information will be accessed only by the researcher and research assistant only.

**Right to refusal or withdraw:** You have the full right to refuse from participating in this research. Your refusal will not affect you from getting any kind of health related service.

**Person to contact:** If you want to know more information, you can contact;

**Address of the principle investigator**

Mr Moges Gashaw (Bsc, Msc candidate)

Department of physiotherapy, UOG

Cell phone: +251924509390

If you agree to participate in the study described above in the following question below

Yes ---------Continue administering interview

No -----------Give thanks to the participant and proceed to the next participant

Signature of the Supervisor and data collector

Name of data collector ---------------------------Signature----------------Date---------------

Supervisor’s name ------------------------------Signature ----------------Date--------------

***THANK YOU VERY MUCH FOR BEING PART OF THIS STUDY***

## *Instruction to data collectors: Please read the questions aloud in a quiet room and request the participant to repeat the question to you again, to assure good reception of the question.*

## English version Questionnaire

| **Survey information** | | | | |
| --- | --- | --- | --- | --- |
|  |  | | **Response** | **Cd** |
| **1** | Consent has been read and obtained | | 1. Yes 2. No | **I1** |
| **2** | Chart number | |  | **I2** |
| **3** | Interviewers ID | |  | **I3** |
| **4** | Date and time of data collection | |  | **I4** |
| **Socio-demographic questions** | | | | |
|  | **Question** | **Response** | | **Cd** |
| **101** | Sex | 1. Male 2. Female | | **S1** |
| **102** | How old are you? | ................in years | | **S2** |
| **103** | Residence | 1.Urban 2.Rural | | **S3** |
| **104** | What is your highest level of education?  Circle appropriate one | 1. No formal school 2. Primary school  3. Secondary school 4. Diploma  5. Degree& above | | **S4** |
| **105** | What is your religion? | 1. Orthodox 2. Protestants  3. Muslim 4. Others | | **S5** |
| **106** | What is your region? | 1. Amhara2.Tigre  3.Oromo 4.Others | | **S6** |
| **107** | What is your marital status? | 1. Single (not married)  2.Currently married  3. Divorced  4.Widowed  5. Separated | | **S7** |
| **108** | What is /was your occupation? | 1.House wife (for female)  2. Farmer  3. Civil servant  4.Merchant  5.Retired  6. Unemployed  7.other, please specify | | **S8** |
| **109** | What is your monthly income? | .......................in Birr | | **S9** |
| **110** | Height | ………………in cm | | **S10** |
| **111** | Weight | ……………..kg | | **S11** |
| **113** | How would you describe your home environment? | 1. Comfortable to do activities  2. Slippery floor  3.Inadequate lighting  4. Poorly accessible light switch  5.Poor housing facilities | | **S12** |
| **114** | How would you describe your current level of mobility? | 1.Independent with mob aids  2. Independent without mob aids | | **S13** |

| **Behavioral factor** | | | | | Response | | | | | Cd |
| --- | --- | --- | --- | --- | --- | --- | --- | --- | --- | --- |
| **201** | | How would you describe your smoking habit? | | | 1. Non smoker 2. Previous smoker 3. Current smoker | | | | | **B1** |
| **202** | | How would you describe your alcohol habit? | | | 1. Non alcoholic 2. Previously alcoholic 3. Currently alcoholic | | | | | **B2** |
| **204** | | Do you do physical exercise? | | | 1. Yes 2. No | | | | | **B4** |
| **205** | | If yes**B4** how many minutes/week? | | | ………in minutes | | | | | **B5** |
| **Psychosocial factors** | | | | | | | | | | |
| **301** | | 1. Are you worried/concerned/afraid of fall during activities of daily living. 2. If yes, Rate your fear of fall (**FES**) | | | Yes / No  1. not at all concerned 2. Some what concerned 3. very concerned | | | | **P1** | |
| **302** | | Do you have good support from family/friends/relatives? | | | 1. Yes 2. No | | | | **P2** | |
| **303** | | Are you suffering from sleep disturbance? | | | 1. Yes 2. No | | | | **P3** | |
| **304** | | If yes to **P4** how often | | | 1. Daily 2. Frequently  3. Occasionally | | | | **P4** | |
| M**edication and co-morbidity** | | | | | | | | | | |
| **401** | Record the presence of the listed conditions from the medical record of the participants  *(Condition You can select more than one)* | | | | | 1. HTN 2. DM 3. Depression  4. Cardio-Vas Disease  5. Chr-lung disease  6. Cancer 7. Foot problem  8. MSK disorders  9. Urogenital disorders  10. Psychiatric disorder  11.if other please specify______________________________ | | | **M1** | |
| **402** | Do you currently take medications? | | | | | 1. Yes 2. No | | | **M2** | |
| **403** | If yes to M3, How many drugs do you take in a day? | | | | | …….……in number | | | **M3** | |
| **404** | Do you suffer from poor urine control? | | | | | 1. Yes 2. No | | | **M4** | |
|  |  | | | | |  | | |  | |
| **Vision related questions** | | | | | |  | | | | |
| **501** | Presenting visual acuity in Snellen chart- *to be filled by ophthalmic nurse* | | 1. Right eye: 2. Left eye: | | | | | **V1** | | |
| **502** | Presenting categories of severity of visual impairment  *[Tick appropriate box]* | | 1. Mild VI in one, normal vision in other | | | |  | **V2** | | |
|  |  |  | 2. Sever VI in one, normal vision in other | | | |  |  |  |  |
|  |  |  | 3. Mild VI in both eye | | | |  |  |  |  |
|  |  |  | 4. Severe VI in one eye & mild in the other eye | | | |  |  |  |  |
|  |  |  | 5. Sever visual impairment in both eyes | | | |  |  |  |  |
| **503** | The cause of visual impairment; from medical records | | 1.Cataract 2.Glaucoma  3.Maculopathy 4.Diabetic Retinopathy  5.Congenital 6. Refractive error  6. Other Specify……………………………………… | | | | | **V3** | | |
| **Fall related questions** | | | | | | | | | | |
| **601** | Did you sustain any falls in the previous 12 months? | | | 1. Yes 2. No, ***If no end*** | | | | **F1** | | |
| **602** | If yes question **F1**. Did you seek medical attention after sustaining falls in previous year? | | | 1. Yes 2. No | | | | **F2** | | |
| **603** | Type of assistance at the time of fall | | | 1. Unassisted 2. Assisted | | | | **F3** | | |
| **604** | What type of injuries did you suffer from the fall?  *[More than one option possible}* | | | 1.Bruises/abrasions/skin tear  2. Bleeding  3. Fracture/dislocation [Specify……………………….]  4. Back pain  5. Loss of consciousness  6. Head injury  Other specify…………………………………….… | | | | **F4** | | |
| **605** | How many falls did you sustain in the previous 12 months? | | | 1. One B. Two 2. Three D. Frequently | | | | **F5** | | |
| **606** | How did u fall? [Fall type] | | | 1.I Tripped 2.I slipped  3.I lost my balance 4.My legs gave away  5.I feel faint 6.I feel dizzy  7. Asleep or sedated8. Bad support  9.I am not sure | | | | **F6** | | |
| **607** | Where have you fallen?  [Place of fall] | | | 1.Inside my home   1. Get out of abed 2. Get out of chair 3. Using shower/bath/toilet 4. Walking up/down stair   2. Away from home   1. In public building 2. Get out of vehicles 3. In other person home | | | | **F7** | | |
| **608** | Activity at time of fall  [tick one] | | | 1. Ambulating 2. Getting out of bed 3. Sitting down/standing up 4. Using toilet 5. Standing or sitting 6. Reaching for object 7. Getting into bed 8. Dressing/undressing 9. Unknown | | | | **F8** | | |
| **609** | At what time did you fall? | | | 1. Night time(6pm-5:59am)  2. Early morning(6am-10:59am)  3. Mid-day(11am-2:59pm)  4. After noon(3pm-5:59pm) | | | | **F9** | | |
