# Supplementary material for: Self-reported fall and associated factors among adult people with visual impairment in Gondar, Ethiopia: a cross-sectional study
Source: BMC Public Health. 2020 Apr 15;20:498. doi: 10.1186/s12889-020-08628-2 (PMC7161228; doi:10.1186/s12889-020-08628-2)
Supplement: Supplementary file 3 — Additional file 3. Additional table for univariate analysis of visual characteristics and fall, frequency distribution of fall-related injuries. [file 12889_2020_8628_MOESM3_ESM.docx]

**Appendix 2**

Table 1 Univariate analysis of visual characteristics variables with among adults with visual impairment in Gondar specialized referral hospital, Ethiopia (n=328)

| **Visual related Variables** | **Self-reported fall** | | **Univariate**  **COR (95%CI)** |
| --- | --- | --- | --- |
|  | Yes | No |  |
| **Cause of Visual impairment**  Cataract  Glaucoma  ARM  Diabetic retinopathy  URE  Others eye disease | 21  37  8  10  8  4 | 13  116  17  3  5  40 | 1.97 (0.70-5.54)  2.91 (0.7-12.18)  2.06 (0.30-14.13)  0.97 (0.26-3.69)  0.62 (0.12-3.15)  1.00 |
| **Severity of visual impairment**  Mild  Moderate  Severe | 18  39  31 | 115  93  32 | 1.00  0.16(0.08-0.33) *  0.43(0.23-0.79) * |
| **Visual impairment (eye involved)**  One eye  Both eyes | 06  82 | 70  170 | 1.00  5.62 (2.35-13.49)* |

**Table 2 Frequency distribution of fall-related injuries among adults with visually impaired fallers (n 88) in Ethiopia**

| **Fall-related injuries** | **Frequency** | **Percent** |
| --- | --- | --- |
| Skin abrasion | **63** | **71.6** |
| Bleeding | **08** | **9.1** |
| Fractures | **17** | **19.3** |
| Dislocations | **11** | **12.5** |
| Head injury | **8** | **9.1** |
| Back pain | **13** | **14.8** |
| Loss of consciousness | **01** | **1.1** |
| Other injuries (chest, abdomen, groin) | **03** | **3.4** |
